# Comparative Evaluation of Standard RT-PCR Assays and Commercial Real-Time RT-PCR Kits for Detection of Lassa Virus

**DOI:** 10.1128/spectrum.05011-22

**Published:** 2023-03-28

**Authors:** Xue-Lian Luo, Xiu-Dan Zhang, Bei-Jie Li, Tian Qin, Zhi-Jie Cao, Qian-Jin Fan, Jing Yang, Dong Jin, Shan Lu, Ya-Yun Zheng, Xue-Fang Xu, Ji Pu, Jianguo Xu

**Affiliations:** a State Key Laboratory of Infectious Disease Prevention and Control, National Institute for Communicable Disease Control and Prevention, Chinese Center for Disease Control and Prevention, Changping, Beijing, China; b Department of Epidemiology, School of Public Health, Shanxi Medical University, Taiyuan, Shanxi Province, China; c Institute of Public Health, Nankai University, Tianjin, China; d Department of Laboratorial Science and Technology & Vaccine Research Center, School of Public Health, Peking University, Beijing, China; Johns Hopkins Medicine

**Keywords:** Lassa virus, RT-PCR, real-time RT-PCR, specificity, sensitivity

## Abstract

Lassa virus (LASV) is a causative agent of hemorrhagic fever epidemic in West Africa. In recent years, it has been transmitted several times to North America, Europe, and Asia. Standard reverse transcription (RT)-PCR and real-time RT-PCR are extensively used for early detection of LASV. However, the high nucleotide diversity of LASV strains complicates the development of appropriate diagnostic assays. Here, we analyzed LASV diversity clustered with geographic location and evaluated the specificity and sensitivity of two standard RT-PCR methods (GPC RT-PCR/1994 and 2007) and four commercial real-time RT-PCR kits (namely, Da an, Mabsky, Bioperfectus, and ZJ) to detect six representative LASV lineages using *in vitro* synthesized RNA templates. The results showed that the GPC RT-PCR/2007 assay had better sensitivity compared to the GPC RT-PCR/1994 assay. The Mabsky and ZJ kits were able to detect all RNA templates of six LASV lineages. Contrastingly, the Bioperfectus and Da an kits failed to detect lineages IV and V/VI. The limit of detection for lineage I with the Da an, Bioperfectus, and ZJ kits were significantly higher than that of the Mabsky kit at an RNA concentration of 1 × 10^10^ to 1 × 10^11^ copies/mL. The Bioperfectus and Da an kits detected lineages II and III at an RNA concentration of 1 × 10^9^ copies/mL, higher than that of the other kits. In conclusion, the GPC RT-PCR/2007 assay and the Mabsky kit were suitable assays for the detection of LASV strains based on good analytical sensitivity and specificity.

**IMPORTANCE** Lassa virus (LASV) is a significant human pathogen causing hemorrhagic fever in West Africa. Increased traveling around the world raises the risk of imported cases to other countries. The high nucleotide diversity of LASV strains clustered with geographic location complicates the development of appropriate diagnostic assays. In this study, we showed that the GPC reverse transcription (RT)-PCR/2007 assay and the Mabsky kit are suitable for detecting most LASV strains. Future assays for molecular detection of LASV should be based on specific countries/regions along with new variants.

## INTRODUCTION

Lassa virus (LASV) causes Lassa fever (LF), a viral hemorrhagic fever in humans with an estimated 5,000 deaths and 100,000 to 300,000 infections annually in West Africa (1, 2). LASV belongs to the family *Arenaviridae* and is a bipartite, single-stranded ambisense RNA virus. The viral genome encodes four proteins: nucleoprotein (NP) and glycoprotein precursor (GPC) on the small (S) segment; and RNA-dependent RNA-polymerase (L) and matrix RING zinc-finger protein (Z) on the large (L) segment (3, 4). LASV presents a high-level nucleotide diversity for the L (32%) and S (25%) segments between strains (5). Based on the geographic location with clustering of strains, LASVs were divided into at least six major LASV lineages: lineages I to III are present in different regions of Nigeria; the largest lineage, IV, circulates in Sierra Leone, Guinea, and Liberia; lineage V is identified in Mali and Ivory Coast; and lineage VI originates from Togo (5–8). Although LF is endemic in West Africa, imported cases have been reported in North America, Europe, and Asia (9–11).

A laboratory test is required to rapidly distinguish LF from other febrile disease with similar clinical symptoms (12). Due to the highly sensitive and specificity, and the materials with the inactivated specimens, PCR-based assays such as standard reverse transcription (RT)-PCR and real-time RT-PCR were prioritized for the detection of LASV. GPC RT-PCR/1994, a standard RT-PCR assay, targets the downstream GPC gene and has been used for routine LF diagnostics (13). In 2007, based on the GPC RT-PCR/1994 assay, a new RT-PCR assay (GPC RT-PCR/2007) was developed to improve sensitivity for the detection of Liberian and Nigerian Lassa virus strains (14). Recently, a real-time RT-PCR assay targeting the L segment was shown to detect LASV lineages I to IV (15). Various commercial real-time RT-PCR kits are now available in the Chinese market, but the sensitivity and specificity of these different kits have not been adequately evaluated. In the present study, we analyzed the diversity of the epidemic LASV strains and compared two standard RT-PCR assays (GPC RT-PCR/1994 and 2007) and four commercial real-time RT-PCR kits (namely, Da an, Mabsky, Bioperfectus, and ZJ) for the detection and quantification of six representative LASV lineages I to VI.

## RESULTS

### Sequence diversity of LASV strains in countries of endemicity.

**FIG 1:**
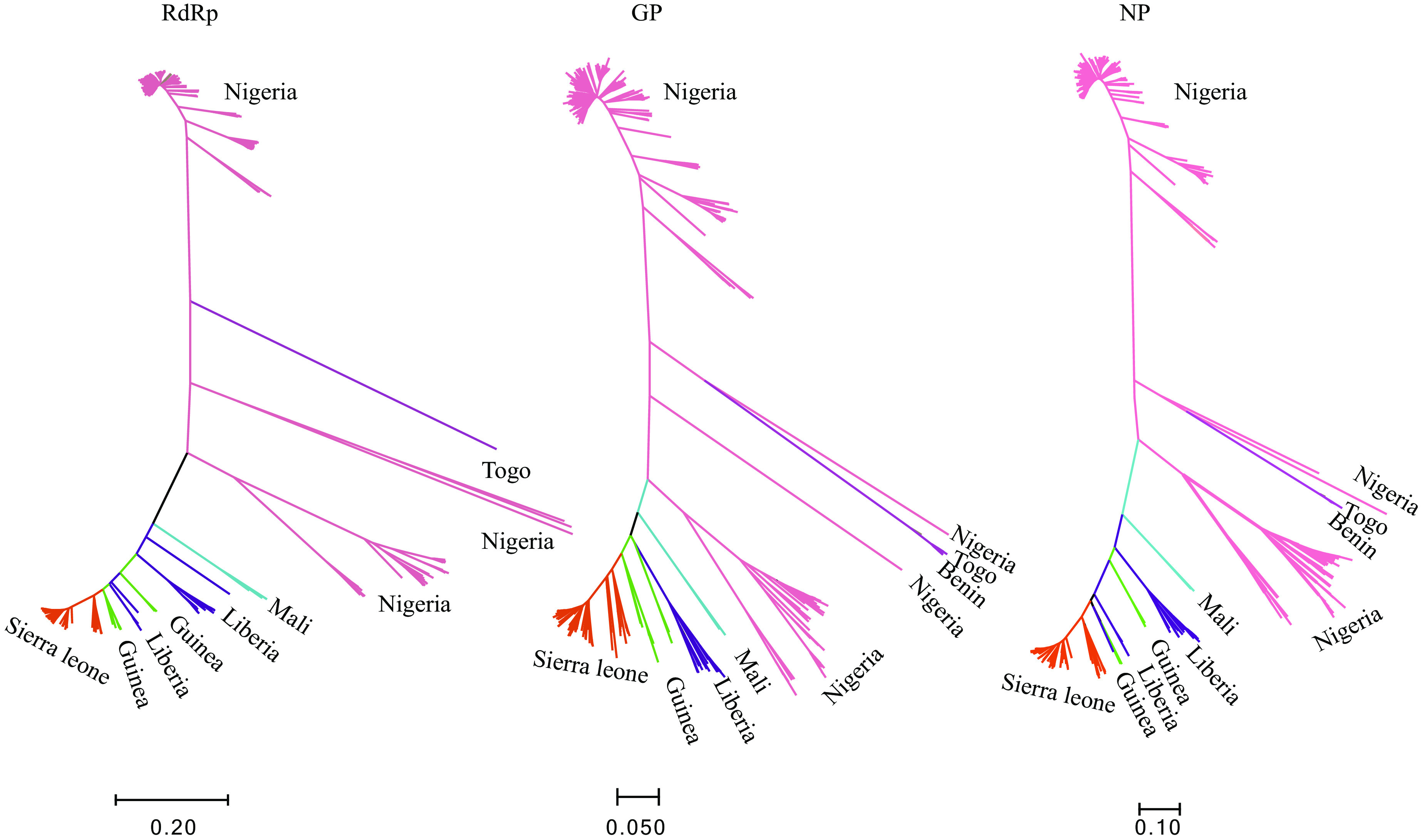
Phylogenetic trees based on RDRP, glycoprotein (GP), and nucleoprotein (NP) genes of Lassa virus (LASV) strains.

The complete sequences of LASV RDRP, GP, and NP genes were aligned by MAFFT software, and phylogenetic trees are shown in Fig. 1. LASV strains showed a great diversity in countries of endemicity and clustered with geographic location, especially those of Nigeria and Sierra Leone. The nucleotide identities of LASV strains were 71% and 77% for the L and S segments, respectively. Pairwise distance analysis of LASV strains from different countries of endemicity was then performed (Table 1). The nucleotide diversity of LASV strains from the different countries of endemicity ranged from 0.78 to 0.82,0.77 to 0.80, and 0.70 to 0.75 for GP, NP, and RdRp, respectively (Table 1). Such high nucleotide diversity between LASV strains in West Africa might hurdle the development of the diagnostic assays.

**TABLE 1:** Estimated evolutionary divergence between Lassa virus strains from different countries of endemicity^*a*^

Protein	Internal/external^*b*^						
	Benin	Guinea	Liberia	Mali	Nigeria	Sierra Leone	Togo
Glycoprotein	0.97/0.81	0.88/0.81	0.87/0.82	0.87/0.80	0.88/0.78	0.92/0.81	1/0.80
Nucleoprotein	0.97/0.80	0.87/0.80	0.87/0.80	0.87/0.80	0.88/0.77	0.92/0.80	1/0.80
Polymerase	1^*c*^/0.73	0.83/0.74	0.84/0.74	0.85/0.73	0.86/0.73	0.91/0.75	1/0.70

a The distance values represent the means.

b The values represent the distance among strains from the same country of endemicity and different countries of endemicity, respectively.

c Because of few sequences, the distance value was 1.

### Sensitivity and specificity of two standard RT-PCR assays.

**FIG 2:**
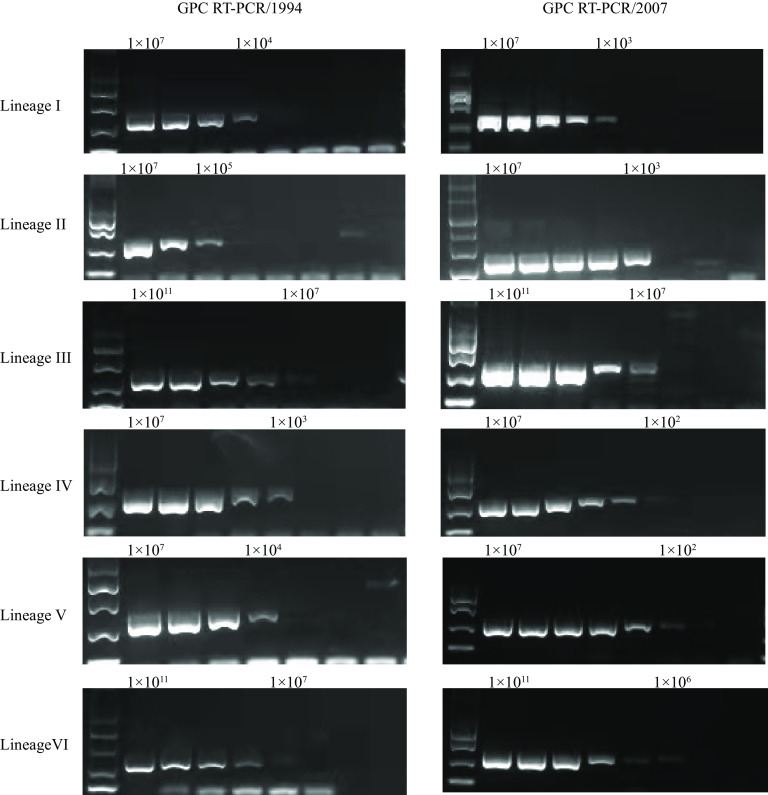
Sensitivity of the glycoprotein precursor (GPC) reverse transcription (RT)-PCR/1994 and 2007 assays for detection of LASV lineages I to VI based on RNA templates.

To compare the sensitivity and specificity of standard RT-PCR assays (GPC RT-PCR/1994 and 2007) (13, 14), six positive controls were generated using *in vitro* synthesized RNA templates from six representative LASV lineages (Table 2). All positive controls (1 × 10^12^ copies/mL) were diluted in 10-fold steps and amplified in parallel (Fig. 2). As shown in Fig. 2, all lineages were detected by both assays. In addition, the limits of detection (LODs) for lineages III and VI were much higher than that of other lineages in both assays at an RNA concentration of 1 × 10^7^ copies/mL (Fig. 2). This indicates that the primer pairs used in the two assays did not bind to the lineages III and VI sufficiently. The LOD of GPC RT-PCR/2007 assay was equal (lineage III) or lower (other five lineages) than that of the GPC RT-PCR/1994 assay (1 to 2 orders of magnitude; Fig. 2). These results suggested that the GPC RT-PCR/2007 assay performed as well as or better than the GPC RT-PCR/1994 assay.

**TABLE 2:** Strain names, GenBank accession numbers, and target regions of positive controls

Strain	Lineage	GenBank accession no. (L segment/S segment)	Target region		
			Standard RT-PCR/Da an kit/ZJ kit	Mabsky kit	Bioperfectus kit
Pinneo	I	KM822127 (L)/AY628207 (S)	1-349 (S)	3490-3661 (L)	1907-2002 (S)
ISTH1121-NIG-2012	II	KM821944 (L)/KM821945 (S)	1-354 (S)	3420-3590 (L)	1934-2034 (S)
Nig08-A19	III	GU481073 (L)/GU481072 (S)	1-340 (S)	3386-3565 (L)	1907-2007 (S)
Josiah	IV	HQ688674 (L)/HQ688672 (S)	1-360 (S)	3407-3578 (L)	1912-2012 (S)
Ouoma-R123	V	KF478764 (L)/KF478768 (S)	1-339 (S)	2904-3080 (L)	1912-2012 (S)
Togo/2016/7082	VI	KU961972 (L)/KU961971 (S)	1-340 (S)	3440-3611 (L)	1912-2012 (S)

**FIG 3:**
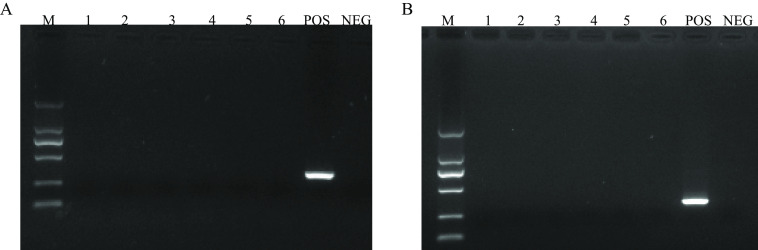
Specificity of the GPC RT-PCR/1994 (A) and GPC RT-PCR/2007 (B) assays for detection of other viruses such as Rift Valley fever virus, Dengue virus types 1 and 3, West Nile virus, and hepatitis C and B virus. NEG, negative (H_2_O); POS, *in vitro* synthesized RNA of LASA strain Pinneo.

Nucleic acids of other hemorrhagic fever viruses, including Rift Valley fever virus, Dengue virus types 1 and 3, and West Nile virus, were obtained from the United Nations Secretary-General’s Mechanism (UNSGM) project RefBio virus EQAE (2021) and used to test the specificity of both assays. In addition, other potential viruses in the blood of patients in the tropics, such as hepatitis C virus and hepatitis B virus, were also tested. No positive bands or signals were detected with either assay (Fig. 3).

### Sensitivity of four commercial real-time RT-PCR kits.

Four commercial real-time RT-PCR kits were used for the detection of LASV RNA following the manufacturer’s instructions (see the Materials and Methods section). The Bioperfectus kit (~45 min/run) had the shortest run time, whereas the Mabsky (~82 min/run) had the longest run time.

**FIG 4:**
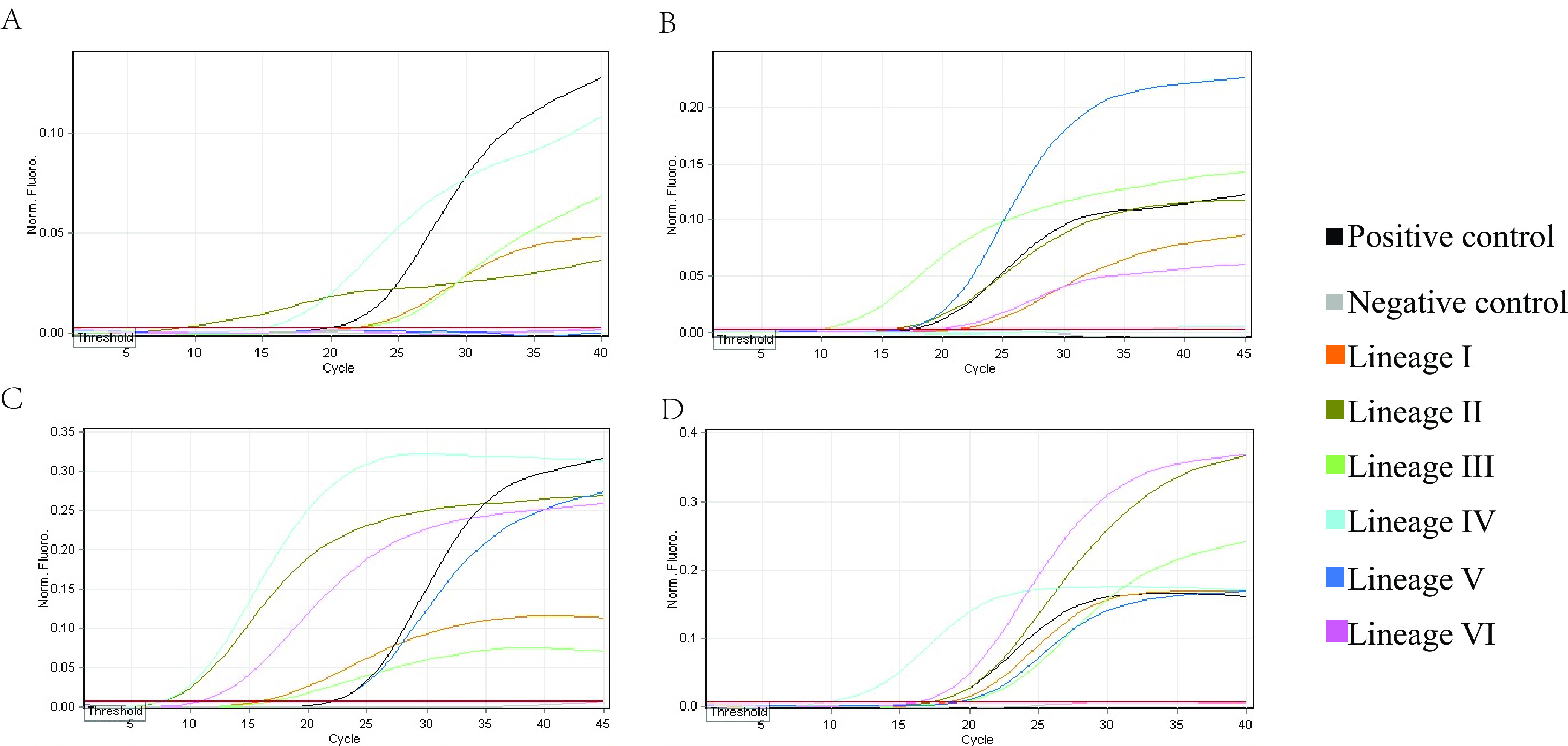
Sensitivity of four commercial real-time RT-PCR kits for detection of LASV lineages I to VI based on RNA templates. (A) Da an kit. (B) Bioperfectus kit. (C) Mabsky kit. (D) ZJ kit.

The target regions of the positive controls in the Mabsky (L segment, ~2,900 to 3,700 nucleotides [nt]) and Bioperfectus (S segment, ~1,900 to 2,040 nt) kits was different from that of standard RT-PCR assays, Da an and ZJ kits (S segment, ~1 to 350 nt) (Table 2). The data showed that the Mabsky and ZJ kits were able to detect all six lineages (Fig. 4C and D). The Bioperfectus kit detected all lineages except lineage IV (Fig. 4B). The Da an kit could only detect four lineages, failing to detect lineages V and VI (Fig. 4A). These results showed that the Bioperfectus and Da an kits had lower sensitivity compared to the Mabsky and ZJ kits, which might result in false negatives.

### Limit of detection for LASV in four commercial real-time RT-PCR.

We next evaluated the LOD of four different commercial real-time RT-PCR kits using LASV lineages I, II, and III. The LOD was determined using 10-fold serial dilutions of positive controls representing LASV lineages I, II, and III. As shown in Table 3, the LOD for lineage I with the Da an, Bioperfectus, and ZJ kits were significantly higher than that of the Mabsky kit at an RNA concentration of 1 × 10^10^ to 1 × 10^11^ copies/mL. The Bioperfectus kit showed much higher LOD against lineage II at an RNA concentration of 1 × 10^9^ copies/mL, compared to other kits (Table 3). For lineage III, the LOD of the Da an kit was much higher than that of other kits at an RNA concentration of 1 × 10^9^ copies/mL (Table 3). These results suggested that the Mabsky kit had the best sensitivity for detection of LASV lineages I, II, and III.

**TABLE 3:** Limits of detection for RNA templates representative of Lassa virus lineages I to VI^*a*^

RNA (copies/mL)		Mean Ct ± SD^*b*^			
		Da an	Mabsky	Bioperfectus	ZJ
Lineage I	1 × 10^12^	29.7 ± 0.2	ND	29.5 ± 0.1	32.1 ± 0.2
	1 × 10^11^	32.1 ± 0.3	ND	32.2 ± 0.2	36.1 ± 1.1
	1 × 10^10^	35.4 ± 0.7	12.4 ± 0.2	35.0 ± 0.2	—
	1 × 10^9^	—	17.0 ± 0.1	—	ND
	1 × 10^8^	ND	20.3 ± 1.1	ND	ND
Lineage II	1 × 10^11^	ND	ND	31.7 ± 0.1	ND
	1 × 10^10^	ND	ND	33.9 ± 0.3	ND
	1 × 10^9^	ND	ND	36.7 ± 0.1	ND
	1 × 10^8^	22.4 ± 0.3	15.0 ± 0.2	—	22.4 ± 0.1
	1 × 10^7^	25.9 ± 0.3	18.1 ± 0.1	ND	25.2 ± 0.1
	1 × 10^6^	28.7 ± 0.1	22.0 ± 0.3	ND	28.1 ± 0.2
Lineage III	1 × 10^12^	26.7 ± 0.1	ND	ND	16.0 ± 0.2
	1 × 10^11^	29.9 ± 0.1	ND	ND	18.8 ± 1.0
	1 × 10^10^	33.0 ± 0.2	ND	ND	24.0 ± 1.4
	1 × 10^9^	36.7 ± 0.5	13.0 ± 0.4	10.9 ± 0.4	ND
	1 × 10^8^	—	16.6 ± 0.3	14.8 ± 0.5	ND
	1 × 10^7^	ND	20.8 ± 0.8	17.9 ± 0.1	ND

a The table shows the limits of detection for RNA templates representative of Lassa virus lineages I to VI in four commercial real-time reverse transcription (RT)-PCR kits. —, negative; ND, not done.

b There were three replicates in each concentration.

## DISCUSSION

LASV causes an acute and potentially fatal hemorrhagic LF and has been listed as a priority pathogen of epidemic potential by the World Health Organization Global Observatory on Health Research and Development (WHO R&D) (16, 17). Despite LASV being endemic in several West African countries, the “spill-over” risk of this disease to other places through traveling has always existed (9–11). Virus isolation, antibody-based assays, and PCR-based assays are commonly used methods for LASV diagnostics (18). PCR-based assays have an advantage in the sensitivity, specificity, and safety with inactivated samples and thus have been used for surveillance and early detection of LASV (12–14, 16, 18).

Appropriate development of PCR-based assays is complicated by Lassa virus genetic diversity (12, 18). The high nucleotide diversity of LASV strains in West Africa (Fig. 1; Table 2) hindered the design of primer/probe pairs for the detection of all LASV lineages (14, 15). Usually, the combined application of multiple primer/probe pairs in the assay decreases primer-template mismatches. On the other hand, due to the geographic cluster displayed by LASV diversity, the future strategy could be the design of specific assays based on specific countries/regions (12).

The GPC RT-PCR/1994 and GPC RT-PCR/2007 assays have been widely used in laboratories for detection of LASV (12, 19). A reference laboratory participating in an external quality Assessment (EQA) study for the molecular diagnosis of LASV recommends GPC RT-PCR/2007 for LASV detection. This is the assay most commonly used, with a good detection rate and the ability to detect all described LASV strains (19). In this study, both RT-PCR assays could detect all six LASV lineages using *in vitro* synthesized RNA templates. Overall, the sensitivity of GPC RT-PCR/2007 assay for detection of LASV lineages I to VI was better than that of GPC RT-PCR/1994 assay (Fig. 2), which was consistent with previous reports (14, 19). In both RT-PCR assays, there is a potential for false negatives in detection of LASV lineages III and VI.

We also evaluated four commercial real-time RT-PCR kits in the Chinese market for the detection of LASV RNAs of six representative lineages. Mabsky and ZJ kits detected all six LASV lineages, whereas the Bioperfectus kit missed lineage IV, and the Da an kit missed lineages V and VI. It might be speculated that the inappropriate primer/probe will affect sensitivity and amplification efficiency. With the emergence of novel LASV strains, the primer/probe needs to be continuously optimized to ensure effective detection of newly emerging strains.

LF cases have not been reported in China, which limited our access to clinical samples or isolated viruses for this study. Therefore, the major limitations of the current study included the data from six synthesized RNA controls representing LASV lineages I to VI and a limited number of reagent batches, which might influence true analytical performance. In future, the clinical samples should be used to confirm the nucleic acid amplification kits for LASV detection.

In conclusion, the GPC RT-PCR/2007 assay and the Mabsky kit showed excellent performance for RNA detection of LASV with high sensitivity and specificity. For PCR-based LASV detection, we must remain vigilant on the variation of virus sequence and develop pangenotypic assays or genotype-specific assays based on countries/regions.

## MATERIALS AND METHODS

### Data collection.

The sequences of LASV RDRP, GP, and NP genes were download from the National Center for Biotechnology Information (NCBI) (http://www.ncbi.nlm.nih.gov/GenBank). After removal of low-quality data, a total of 467, 626, and 630 complete sequences of LASV RDRP, GP, and NP genes, respectively, were used for phylogenetic and distance analysis.

### Phylogenetic analysis.

Sequence alignment was performed using MAFFT version 7 with the L-INS-I algorithm (20). Phylogenetic trees were inferred using the maximum likelihood method implemented in PhyML version 3.0 with the GTR nucleotide substitution model and a Subtree Pruning and Regrafting topology searching algorithm (21). Analyses were performed on a bootstrapped data set (100 replicates). The estimates of evolutionary divergence between sequences was calculated by MEGA version 7 (22).

### Generation of positive controls.

LASV strains (Pinneo, ISTH1121-NIG-2012, Nig08-A19, Josiah, Ouoma-R123 and Togo/2016/7082) belonging to lineages I to VI, respectively, were randomly selected as six positive templates (5). The detail information of these positive templates, including strain name, GenBank accession number, and amplification region is listed in Table 2. The DNA sequences in target region were synthesized by Sangon Biotech (Shanghai, China) Co., Ltd., and then cloned into T7 polymerase expression vector pGEM-T (Invitrogen, USA). The pGEM-T plasmids were used as a template to be *in vitro* transcribed into RNA according to T7 *in vitro* transcription kit (Promega, USA). The RNA was purified with RNeasy columns (Qiagen, Germany) and quantified using Qubit 3 (Invitrogen, USA) as final positive controls.

### Standard RT-PCR.

Both GPC RT-PCR/1994 and 2007 assays were performed according the published protocol using PrimeScript One Step RT-PCR kit (TaKaRa, Japan), with some modification (13, 14). Briefly, a 25-μL mixture contained 5 μL RNA, 20 μM primer pairs 36E2/80F2 (5′-ACCGGGGATCCTAGGCATTT-3′/5′-ATATAATGATGACTGTTGTTCTTTGTGCA-3′) (for GPC RT-PCR/1994 assay) and 36E2/LVS-339-rev (5′-ACCGGGGATCCTAGGCATTT-3′/5′-GTTCTTTGTGCAGGAMAGGGGCATKGTCAT-3′) (for GPC RT-PCR/2007 assay), 12.5 μL 2× RT-PCR buffer, and 1 μL enzyme. The cycling conditions were 50°C for 30 min and 94°C for 2 min, followed by 40 cycles of 94°C for 30 s, 52°C for 30 s, and 72°C for 30 s. The mixture was set up on ice and placed into a Labcycler (Sensoquest, Germany).

### Quantitative RT-PCR (qRT-PCR).

Four commercial kits for the detection of LASV RNA (real-time RT-PCR method) were compared, including Da an (Guangzhou Da An Biotechnology Co., Ltd.), Mabsky (Shenzhen MABSKY Bio-Tech Co., Ltd.), Bioperfectus (Jiangsu Bioperfectus Technologies Co., Ltd.), and ZJ (Shanghai ZJ Bio-Tech Co., Ltd.). All assays contained specific primers and TaqMan probes targeting the different regions (Table 2) and were performed in a Rotor-Gene Q (Qiagen, Germany) according to the respective protocol. Briefly, amplifications were carried out in 25-μL reaction mixtures containing 20 μL buffer and enzyme mixture and 5 μL template RNA. The cycle threshold (Ct) values of 38 (for Da an, Mabsky, and ZJ) and 37 (for Bioperfectus) were positive. For sensitivity assay, serial dilutions of 10^−2^ to 10^−6^ positive control were used to generate calibration curves, respectively.
